# Supplementary material for: Maternal-antenatal attachment in young pregnant women: Social support, mentors, and fear of childbirth
Source: PLoS One. 2025 Jul 2;20(7):e0319449. doi: 10.1371/journal.pone.0319449 (PMC12221006; doi:10.1371/journal.pone.0319449)
Supplement: S1 Table — (DOCX) [file pone.0319449.s001.docx]

**S1 Table. MAAS Items with Response Options.**

| Item | Answer Options |
| --- | --- |
| Over the past two weeks, I have thought about or been preoccupied with the baby inside me. | 1 = Almost all the time  2 = Very frequently  3 = Frequently  4 = Occasionally  5 = Not at all |
| Over the past two weeks, when I have spoken about or thought about the baby growing inside me I felt emotional. These feelings were: | 1 = Very weak or non-existent  2 = Fairly weak  3 = In between strong and weak  4 = Fairly strong  5 = Very strong |
| Over the past two weeks, my feelings about the baby growing inside me have been: | 1 = Very positive  2 = Mainly positive  3 = Mixed positive and negative  4 = Mainly negative  5 = Very negative |
| Over the past two weeks, I have had the desire to read about to get information about my growing baby. This desire is: | 1 = Very weak or non-existent  2 = Fairly weak  3 = In between strong and weak  4 = Fairly strong  5 = Very strong |
| Over the past two weeks, I have been trying to picture in my mind what the growing baby actually looks like in my belly: | 1 = Almost all the time  2 = Very frequently  3 = Frequently  4 = Occasionally  5 = Not at all |
| Over the past two weeks, I think of the baby inside me mostly as: | 1 = A real little person with special characteristics  2 = A baby like any other baby  3 = A human being  4 = A living thing  5 = A thing not yet really alive |
| Over the past two weeks, I have felt that the baby inside me is dependent on me for its well-being: | 1 = Totally  2 = A great deal  3 = Moderately  4 = Slightly  5 = Not at all |
| Over the past two weeks, I have found myself talking to my baby when I am alone: | 1 = Not at all  2 = Occasionally  3 = Frequently  4 = Very frequently  5 = Almost all the time I am alone |
| Over the past two weeks, when I think about (or talk to) my baby inside me, my thoughts: | 1 = Are always tender and loving  2 = Are mostly tender and loving  3 = Are a mixture of both tenderness and irritation  4 = Contain a fair bit of irritation  5 = Contain a lot of irritation |
| The picture in my mind of what the baby at this stage actually looks like inside my belly is: | 1 = Very clear  2 = Fairly clear  3 = Kind of clear  4 = Not very clear at all  5 = I have no idea at all |
| Over the past two weeks, when I think about the baby inside me I get feelings that are: | 1 = Very sad  2 = Moderately sad  3 = A mixture of happiness and sadness  4 = Moderately happy  5 = Very happy |
| Some pregnant women sometimes get so irritated by the baby inside them that they feel like they want to hurt or punish it: | 1 = I can't imagine I would ever feel like this  2 = I can imagine I might feel like this, but I never actually have  3 = I have felt like this once or twice myself  4 = I have occasionally felt like this myself  5 = I have often felt like this myself |
| Over the past two weeks, I have felt: | 1 = Very emotionally distant from my baby  2 = Moderately emotionally distant from my baby  3 = Not particularly emotionally close to my baby  4 = Moderately close emotionally to my baby  5 = Very close emotionally to my baby |
| Over the past two weeks, I have taken care with what I eat to make sure the baby gets a good diet: | 1 = Not at all  2 = Once or twice when I ate  3 = Occasionally when I ate  4 = Quite often when I ate  5 = Every time I ate |
| When I first see my baby after the birth, I expect I will feel: | 1 = Intense affection  2 = Mostly affection  3 = Some affection  4 = Some negative feelings  5 = Mostly negative feelings |
| When my baby is born, I would like to hold the baby: | 1 = Immediately  2 = After it has been wrapped in a blanket  3 = After it has been washed  4 = After a few hours, when things settle down  5 = The next day |
| Over the past two weeks, I have had dreams about the pregnancy or baby: | 1 = Not at all  2 = Occasionally  3 = Frequently  4 = Very frequently  5 = Almost every night |
| Over the past two weeks, I have found myself feeling or rubbing with my hand, the outside of my stomach where the baby is: | 1 = A lot of times each day  2 = At least once per day  3 = Occasionally  4 = Once only  5 = Not at all |
