# Supplementary material for: Maternal-antenatal attachment in young pregnant women: Social support, mentors, and fear of childbirth
Source: PLoS One. 2025 Jul 2;20(7):e0319449. doi: 10.1371/journal.pone.0319449 (PMC12221006; doi:10.1371/journal.pone.0319449)
Supplement: Attachment — Submitted filename: Response to Reviewers.docx [file pone.0319449.s003.docx]

Thank you for the opportunity to revise our manuscript. To ensure that our manuscript meets PLOS ONE's style requirements, I have updated the author byline with appropriate indentation.

The funding statement was updated to include: “Research reported in this publication was supported by the National Institute On Drug Abuse of the National Institutes of Health under Award Number R01DA046401 (NDG). The funders had no role in study design, data collection and analysis, decision to publish, or preparation of the manuscript. There was no additional external funding received for this study.” As requested, this statement can be found in our cover letter.

Data for this analysis do not include any interview transcripts, only responses to an online survey and urine screening results. These data appear in a data repository as an Excel file and no personal details or potentially identifying information has been published. All data analyzed for this paper are identified with participant ID. No personal or identifiable information has been shared publicly.

A caption for my Supporting Information table (S1 Table) is included under the References section with the in-text citation updated to “S1 Table.”

The revised reference list is complete and correct. No cited papers have been retracted. The first author for reference 39 updated her last name, which is reflected in the revised reference list. I added middle initials for authors in reference 56. The order of references remained unchanged.
In response to the reviewer's comments, I edited the name of the article to reflect on more general aspects of investigation into maternal-antenatal attachment.
